# Giant cell myocarditis causing refractory ventricular tachycardia in a pediatric patient

**DOI:** 10.1002/ccr3.1410

**Published:** 2018-02-14

**Authors:** Apurva Panchal, Obehioye Okojie, Brittany Slagle, Ossama Tawfik

**Affiliations:** ^1^ Department of Pediatrics University of Kansas Medical Center Kansas City Kansas; ^2^ Department of Pathology and Laboratory Medicine University of Kansas Medical Center Kansas City Kansas

**Keywords:** Endomyocardial biopsy, giant cell myocarditis, heart transplantation, pediatric patients, ventricular tachycardia

## Abstract

Giant cell myocarditis should be considered in all pediatric patients with refractory ventricular arrhythmia. Endomyocardial biopsy should be obtained to confirm the diagnosis of giant cell myocarditis.

## Introduction

Giant cell myocarditis (GCM) is a rare but aggressive disease seen in middle‐aged adults with the average age of onset at 42.6 years 1. Data are limited in the pediatric population due to the paucity of cases. Patients 19 years and younger comprise just 6% of cases in the multicenter registry for GCM 2.

The etiology of GCM is not well understood. However, animal models and studies in patients suggest autoimmune causes with inappropriate activation of T lymphocytes 3. There is no sex predominance reported, but 20% of patients have other autoimmune disorders 4, 5. Patients commonly present with acute heart failure and less frequently with arrhythmias 1. Treatment is with long‐term immunosuppression, and patients often require implantable cardioverter defibrillators, circulatory support with extracorporeal membrane oxygenation (ECMO) or ventricular assist devices, and heart transplant 1, 5.

Transplant and death appear even more likely outcomes in the pediatric population with almost all patients requiring either transplantation or dying from GCM 2, 6. Even with transplant and immunosuppression, recurrence occurs in nearly 25% of transplanted hearts 1, 6.

## Case Report

A 14‐year‐old boy with a history of attention‐deficit/hyperactivity disorder (ADHD) treated with methylphenidate for last 4 years was presented to an outside hospital with palpitations and chest pain without syncope secondary to hemodynamically stable ventricular tachycardia (VT). His VT was refractory to two attempts of synchronized cardioversion with 1 joule/kg. He was started on an amiodarone infusion and was admitted to our pediatric intensive care unit.

**Figure 1:**
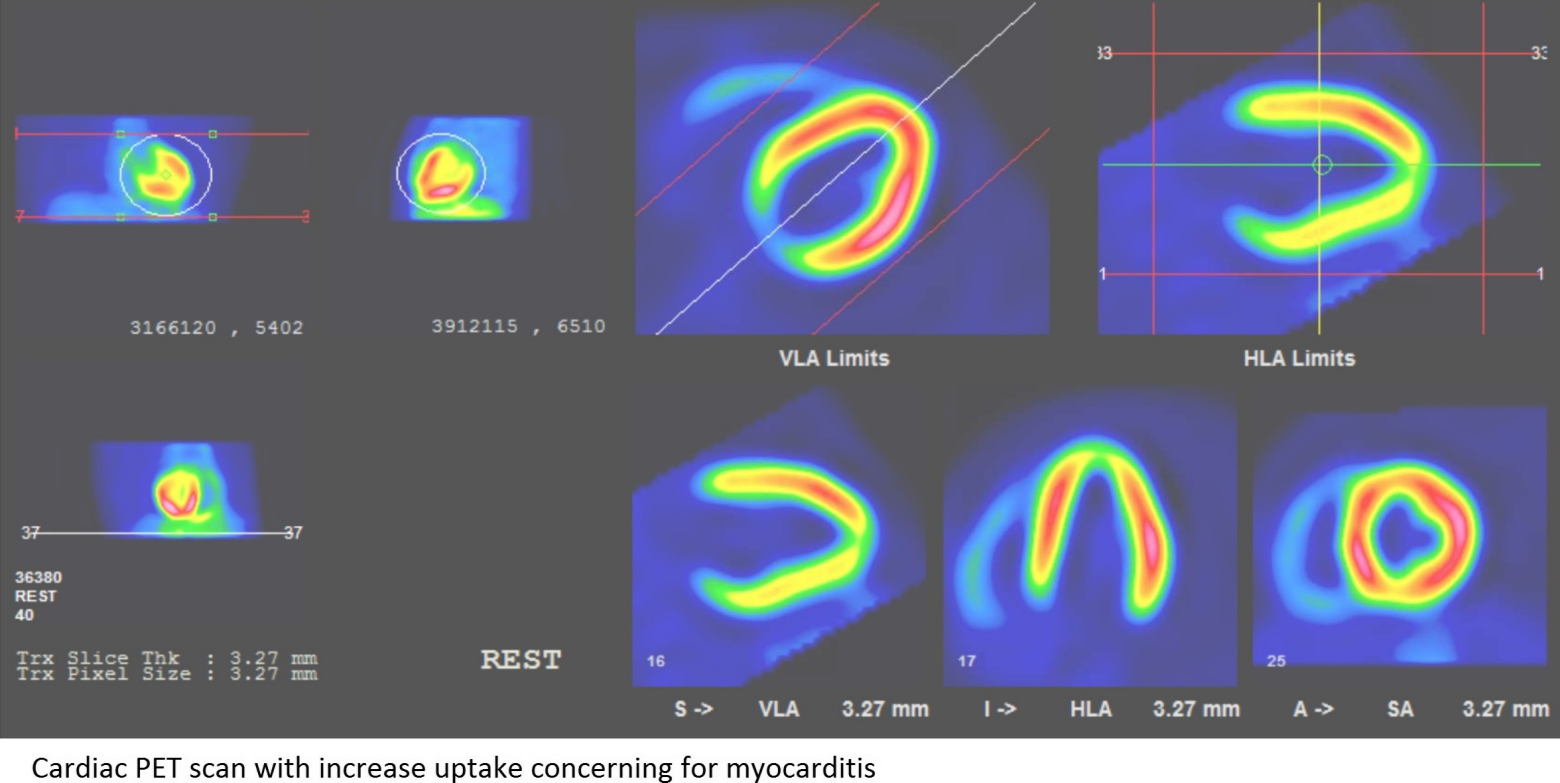
Cardiac positron emission tomography scan demonstrating heterogenous areas of increased uptake involving the mid‐anterolateral wall and mid to basal septal wall concerning for myocarditis.

Upon admission to our pediatric intensive care unit, his initial electrocardiogram (ECG) demonstrated sinus rhythm with multiple intermittent premature ventricular contractions (PVCs) and brief runs of monomorphic VT despite being on amiodarone and discontinuing his methylphenidate. The differentials of arrhythmia associated with cardiomyopathies and myocarditis, secondary to toxins, and genetic diseases were considered. Echocardiogram showed normal heart function with an ejection fraction of 60%. It did not show signs of cardiomyopathies or inflammation. His workup for myocarditis was negative except positive rhino/enterovirus infection. A cardiac magnetic resonance imaging (MRI) study with gadolinium enhancement showed no evidence of cardiomyopathy but showed mildly diminished ejection fraction of 49%. An electrophysiology study was attempted with stimulant medications to induce VT, which showed few nonsustained VT from right outflow tract. However, the accurate mapping for focus was unsuccessful due to short‐lasting VT possibly secondary to his amiodarone exposure. His amiodarone was discontinued, and he was discharged home on a wearable cardioverter‐defibrillator and metoprolol 25 mg daily with a plan to return in a week for a positron emission tomography (PET) scan of the heart and a repeat electrophysiology study with possible ablation. The cardiac PET scan showed heterogeneous increased uptake bilaterally but predominantly left ventricle myocardium (mid‐anterolateral and mid to basal septal walls) consistent with myocarditis (Fig. 1).

**Figure 2:**
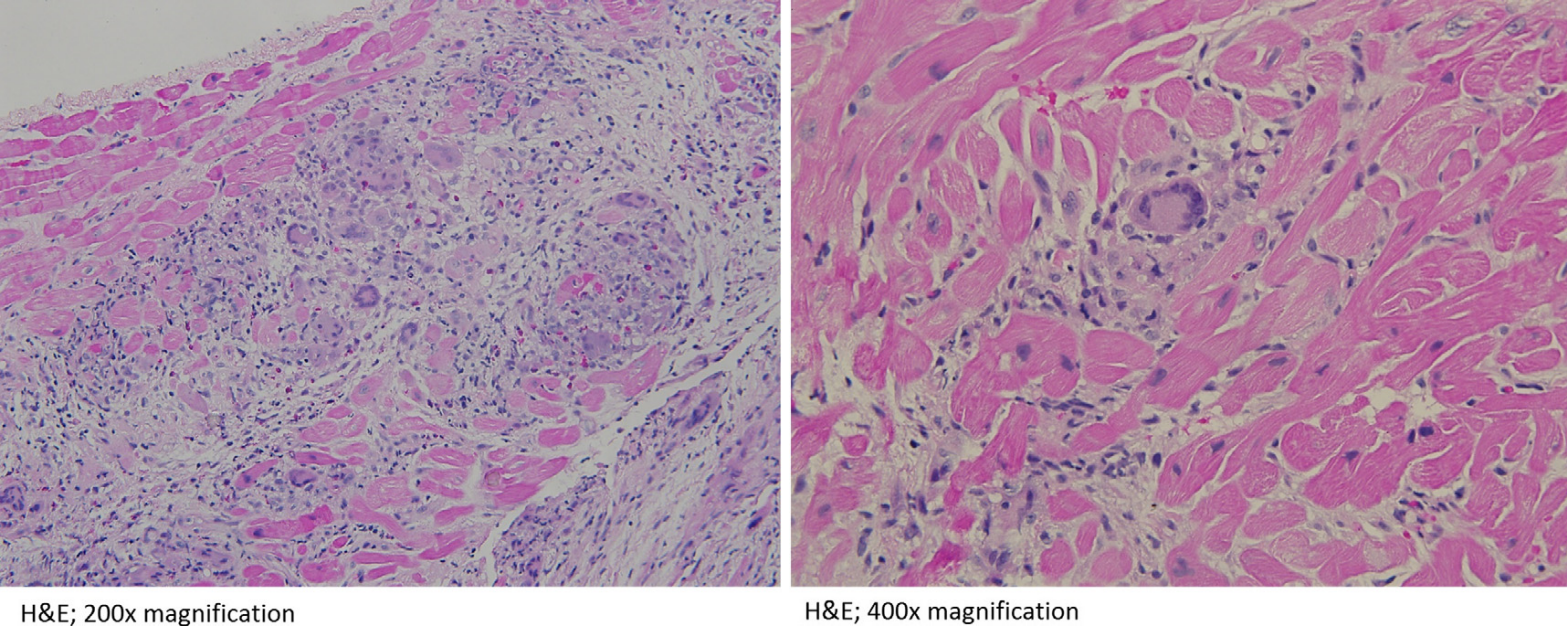
Images of endomyocardial biopsy show extensive lymphoplasmacytic inflammatory infiltrate with eosinophilia and abundant multinucleated giant cells in the representative section of the right ventricle. Occasional myocyte necrosis and interstitial fibrosis are also noted (H&E: Hematoxylin‐Eosin stain).

Repeat electrophysiology study was successful in a reproduction of sustained VT. However, he had VT with ten different morphology coming from various segments of the right and left ventricles. The ablation was aborted, and he underwent an endomyocardial biopsy that revealed giant cell myocarditis (Fig. 2). A rheumatologic workup (except high anti‐nuclear antibody titers) and an infectious disease workup (except positive rhino/enterovirus) were negative. Along with azathioprine and cyclosporine, pulse dose steroid was initiated with 1 g of methylprednisolone daily for 3 days followed by daily prednisone 40 mg. A dual chambered implantable cardioverter‐defibrillator (ICD) was placed transvenously. His metoprolol dose was optimized to 25 mg twice daily. GCM and heart transplantation workup was initiated, and he was eventually listed for heart transplantation.

There is no family history of congenital heart disease, unexplained death, ICD implantation or cardiomyopathy. Maternal grandfather had coronary artery disease, and maternal grandmother had a pacemaker for an unknown reason. The grandparents care for the patient and parents are not involved, their history is unknown.

## Discussion

Saltykow 7 first described nontuberculosis, nonsyphilis associated GCM in 1905. Since then cases of GCM have infrequently been reported. GCM had traditionally been diagnosed during autopsy 1, 2, 3. Recently the endomyocardial biopsy (EMB) has made it possible to diagnose early 3.

Giant cell myocarditis is a rare but devastating disease of unclear etiology affecting mostly middle‐aged adults 1, 2. A rat model and a few other human studies suggested possible T lymphocyte‐mediated granulomatous inflammatory myocarditis as a cause 5. Up to 20% of patients with GCM have other autoimmune conditions such as thyroiditis, inflammatory bowel disease, rheumatoid arthritis 1, 4, 5. Further studies distinguished GCM from sarcoidosis, both histologically and clinically 7. Pathologically, GCM will have diffuse or multifocal infiltrate consisting of lymphocytes and eosinophils with multinucleated giant cells at the periphery and various degree of fibrosis and damaged myocytes 3.

Giant cell myocarditis is a disease of middle‐aged adults with the mean age of 42.6 ± 12.7 years. However, it has been documented in children as young as 6 weeks old 1, 6. Multicenter GCM registry reported only 4 of 63 (6%) patients younger than 19 years with GCM 2. The most common clinical manifestations are congestive heart failure (75%), atrioventricular block (30%), and sustained ventricular tachycardia (15%) 1. A similar trend was seen in the pediatric population with GCM studies by Das et al. 6. Our patient presented with ventricular tachycardia with minimally diminished heart function (ejection fraction of 49% on MRI). Methylphenidate could have caused his ventricular arrhythmia, although it is highly unlikely given the unremarkable first 4 years of its use and now with the sudden onset of VT without a change in the dose. Studies have shown that low expression of interleukin 17 (IL‐17), and tissue necrosis factor alpha (TNF‐*α*) in GCM and other granulomatosis diseases can cause loss of plakoglobin (a desmosomal protein) from intercalated disks 7. Low level of plakoglobin has been linked with arrhythmogenic right ventricular dysplasia, sarcoidosis, GCM, and other disorders associated with ventricular arrhythmia 7.

Unfortunately, echocardiogram and magnetic resonance imaging of heart could not differentiate between GCM and other forms of myocarditis, but they are helpful in differentiating fulminant versus acute myocarditis 6, 7. In our patient, both echocardiogram and MRI failed to diagnose even myocarditis in the early phase of the disease. It was a PET scan that showed moderate myocarditis. Because of concern for heterogeneous myocarditis on PET scan and the fact that he had almost ten different morphological ventricular arrhythmias originating from various sources, EMB was obtained subsequently. In general, EMB has a sensitivity of 70–80% compared to gold standard surgical biopsy 4, 8. In 2013, the European Society of Cardiology recommended EMB in all patients with suspected myocarditis in the presence of one or more clinical features and one or more diagnostic tests (including abnormal ECG) 9. Our patient met the criteria for EMB as he presented with chest pain (one of the clinical features) and an abnormal 12‐lead ECG with VT. The PET scan subsequently confirmed the myocarditis, and EMB revealed the diagnosis of GCM. For those 20% false negative biopsy cases, if the suspicion is high for GCM then a second biopsy is warranted 3, 4.

Eighty‐nine percent of patients with GCM either die from the disease or progress to cardiac transplantation with a median duration of 5.5 months 1. The outcome in the pediatric population with GCM is worse 2, 6. In a review of the 14 reported pediatric GCM cases, fifty percent died before the transplant, and of the fifty percent who survived to transplant, two of them died in the immediate post‐transplant period 6. Immunosuppressive therapy, especially with steroid and cyclosporine (T‐cell suppressor), has shown promising results with partial clinical remission and increased transplant‐free survival has been reported 4. Our patient was also placed on steroid and cyclosporine. He is currently waiting for heart transplantation.

Multicenter registry on GCM in 1997 showed 63 patients with GCM, of which 34 patients received heart transplantation. Of these 34 patients, nine patients died within 3.7 years after heart transplantation 1. Recurrence of GCM in transplanted hearts has been reported multiple times. The same registry reported nine (~25%) cases of recurrence of those 34 patients who received transplantation 1. Similar recurrence rate has been reported by Cleveland Clinics and University of Ottawa Heart Institute 10. Das et al. 6 also reported similar recurrence rate in the pediatric population with GCM who received heart transplantation. Recurrence of GCM in the transplanted heart is not a contraindication for heart transplantation. Despite high recurrence rates, the post‐transplant survival rate is almost 70% at 5 years 6, 10.

In conclusion, GCM should be considered in all pediatric patients with refractory ventricular arrhythmia with or without heart failure. PET scan should be considered when traditional cardiac echocardiogram and MRI fail to diagnose the myocarditis and suspicion are high. Endomyocardial biopsy should follow these imaging studies to confirm the diagnosis of GCM.

## Authorship

All the authors contributed toward the case report by making the substantial contribution. AP, OO, and BS: participated in the medical care of this patient and for the preparation of the manuscript. OT: provided a critical revision of the manuscript, and interpretation of the endomyocardial biopsy.

## Conflict of Interest

All the authors declared no conflict of interest.
